# Advancing guideline quality through country-wide and regional quality assessment of CPGs using AGREE: a scoping review

**DOI:** 10.1186/s12874-023-02101-5

**Published:** 2023-11-30

**Authors:** Marli Mc Allister, Ivan D. Florez, Suzaan Stoker, Michael McCaul

**Affiliations:** 1 Department of Global Health, Division of Epidemiology and Biostatistics, Faculty of Medicine and Health Sciences, Stellenbosch University, Francie Van Zijl Drive TYGERBERG 7505, Cape Town, South Africa; 2 Department of Pediatrics, Faculty of Medicine, University of Antioquia, Medellin, Colombia; 3 School of Rehabilitation Science, McMaster University, Hamilton, Canada; 4 Pediatric Intensive Care Unit, Clinica Las Americas AUNA, Medellin, Colombia

**Keywords:** Clinical practice guidelines, AGREE tools, Quality appraisal, Regional guidelines, Global guidelines, LMICs, Reviews

## Abstract

**Background and objective:**

Clinical practice guidelines (CPGs) are evaluated for quality with the Appraisal of Guidelines for Research and Evaluation (AGREE) tool, and this is increasingly done for different countries and regional groupings. This scoping review aimed to describe, map, and compare these geographical synthesis studies, that assessed CPG quality using the AGREE tool. This allowed a global interpretation of the current landscape of these country-wide or regional synthesis studies, and a closer look at its methodology and results.

**Study design and methods:**

A scoping review was conducted searching databases Medline, Embase, Epistemonikos, and grey literature on 5 October 2021 for synthesis studies using the later versions of AGREE (AGREE II, AGREE-REX and AGREE GRS) to evaluate country-wide or regional CPG quality. Country-wide or regional synthesis studies were the units of analysis, and simple descriptive statistics was used to conduct the analysis. AGREE scores were analysed across subgroups into one of the seven Sustainable Development Goal regions, to allow for meaningful interpretation.

**Results:**

Fifty-seven studies fulfilled our eligibility criteria, which had included a total of 2918 CPGs. Regions of the Global North, and Eastern and South-Eastern Asia were most represented. Studies were consistent in reporting and presenting their AGREE domain and overall results, but only 18% (*n* = 10) reported development methods, and 19% (*n* = 11) reported use of Grading of Recommendations Assessment, Development, and Evaluation (GRADE). Overall scores for domains *Rigor of development* and *Editorial independence* were low, notably in middle-income countries. *Editorial Independence* scores, especially, were low across all regions with a maximum domain score of 46%. There were no studies from low-income countries.

**Conclusion:**

There is an increasing tendency to appraise country-wide and regionally grouped CPGs, using quality appraisal tools. The AGREE tool, evaluated in this scoping review, was used well and consistently across studies. Findings of low report rates of development of CPGs and of use of GRADE is concerning, as is low domain scores globally for *Editorial Independence*. Transparent reporting of funding and competing interests, as well as highlighting evidence-to-decision processes, should assist in further improving CPG quality as clinicians are in dire need of high-quality guidelines.

**Supplementary Information:**

The online version contains supplementary material available at 10.1186/s12874-023-02101-5.

## Introduction

Clinical practice guidelines (CPGs) play an integral part of medical practice, policy, and politics; and wherever possible should be informed by systematic reviews of the best available evidence, while considering benefits and harms. Clinical practice guideline development groups, researchers and clinicians enjoy considerable resources to support and inform guideline development [1], reporting [1–3] and critical appraisal, all developed by a variety of guideline organisations across the globe [4–7]. However, despite the advances of such international repositories, the variation in the quality of CPGs in different countries and regions around the world, speaks to its overall complexity and multiplicity [8–10]. Moreover, development approaches varies (and perhaps rightfully so), especially across countries, ranging from developing guidelines de novo (starting anew) to adopting or adapting CPGs to local contexts [11]. Approaches such as these that rely on using existing high-quality guidelines instead of de novo, provide opportunities to save time and resources, especially relevant in resource-poor settings.

For example, in a study evaluating African hypertension guidelines, recommendations were reported as a mixture of standard treatment guidelines (adapting), WHO guidelines (adopting) and de novo CPG development [12]. Notably, low- and middle-income countries (LMICs) continue to face increasing challenges and complexity, in terms of developing and implementing high quality CPGs; not only regarding capacity and funding, but also an increased burden of diseases (especially infectious diseases), healthcare worker shortages, and weaker health systems [13]. This was recently noted anew in relation to COVID-19 responses and its challenging effect on evidence synthesis groups in LMICs specifically [14], contributing to the call for guideline developers to stratify CPG recommendations more effectively for low-resource settings [15].

Over the last two decades, steady inroads have been made in terms of levelling CPG quality using quality appraisal tools [16]. These tools have become somewhat of a landscape architect, supporting journal editors when reviewing guidelines, underpinning and assessing guideline validity, and allowing the trust placed in guidelines, to be strengthened. The internationally accepted standard for the quality appraisal of CPGs, is the Appraisal of Guidelines for Research and Evaluation (AGREE) tool [17–19], notably the latest AGREE II tool and others (AGREE-REX and -GRS). This tool is comprised of six domains including ‘Scope and purpose’, ‘Stakeholder involvement’, ‘Rigor of development’, ‘Clarity of presentation’, ‘Applicability’, and ‘Editorial independence’. Appraisal of guidelines using these six domains allows CPG developers, researchers, and decision makers to critically evaluate fundamental elements of guideline construction, quality, and implementation ability.

The AGREE tools have been used for a variety of reasons, from appraising individual CPGs for guideline adaptation, to appraising specific grouped CPGs, including: a sample from the National Guideline Clearinghouse when the AGREE-REX tool was developed [20]; several mixed medical topics [16], as well as targeted medical topics [21]. Multiple countries and regions worldwide have assessed their local, national, or regional CPGs with this tool and reported on this in methodological or review synthesis studies [10, 22–24]. Some countries periodically appraise CPGs using AGREE to gauge progression of guideline quality over time [8, 25, 26]. Added to this, studies have presented quality assessments of CPGs regarding specific disciplines, across various regions [12, 27, 28]. However, there is a paucity of evidence that has sought a worldwide overview; evaluating and comparing how studies focused on assessing the quality of guidelines in specific countries and/or regions, exploring whether the CPG quality differ among these jurisdictions.

This scoping review aimed to fill this knowledge gap by describing and mapping national and regional CPG synthesis studies that used the most recent versions of the AGREE tool (i.e., AGREE II, AGREE REX and AGREE GRS) towards unpacking how AGREE is used and reported in guideline assessment studies. This allowed a comprehensive global view of the characteristics of these national and regional synthesis studies; quantity and quality of the included CPGs; and a global and regional evaluation of the six AGREE domain scores. Additionally, it allowed a focus on specific domains *Rigor of development* and *Editorial independence*. These two important domains have been historically considered [20], and again recently indicated [29], as having the most direct effect on overall CPG content quality.

## Methods

This scoping review described the methodology and characteristics of CPG synthesis studies and its included CPGs; and subsequently mapped and compared studies that used later versions of the AGREE tool, to assess CPG quality country-wide and/or regionally. This includes describing the use of the AGREE tool, comparing domain scores, and ascertaining use of the overall assessment. A protocol was developed a priori (Additional file 1: Appendix A) and this study was conducted in accordance with the Joanna Briggs Institute methodology for scoping reviews [30], where results were reported according to the Preferred Reporting Items extension for Scoping Reviews (PRISMA-ScR) [31].

### Search strategy

A predefined search strategy was used to conduct a comprehensive search in the following databases: Embase (Ovid), Medline (Pubmed), Epistemonikos, and grey literature (Web of Science grey literature, greylit.org, and contacting key experts). The search was conducted on 5 October 2021. Studies published in any language were included until full-text stage. The full Medline and Embase search strategies are listed in Additional file 1: Appendix B.

### Eligibility criteria

#### Participants

Secondary research on CPG quality including scoping reviews; methods studies (including meta-epidemiological studies); reviews; systematic reviews of CPGs; and evaluation/analysis of quality of CPGs were considered. Synthesis studies on country-wide, regional, and topical CPGs were considered. Grey literature including thesis, dissertations and unpublished studies were considered. Exclusions based on study types, were international scoping reviews collating different countries into a topical review.

#### Concept

Any guideline synthesis study that used AGREE II, AGREE GRS (Global Rating Scale: a short item tool especially useful when time and resources are limited), and AGREE-REX (designed to evaluate the clinical credibility and implementation of CPGs) were considered. This tool uses six domains with 23 items, each scored 1–7 (strongly disagree to strongly agree) as well as two overall assessments. The overall assessment requires each assessor to firstly rate the overall quality of the guideline (on a scale of 1–7) and secondly to make a judgement as to whether this guideline is recommended for use (i.e., recommended; recommended with modifications; or not recommended). Exclusions included studies that used other tools or scores to appraise the quality of its CPGs.

#### Context

All countries or regions worldwide and their medical specialities and sub-specialities, including allied health and traditional medicine, were considered. Regions according to WHO, United Nation or Sustainable Development Goals (SDG) regional groupings were considered. Only CPGs answering human, health-related questions were considered. Exclusions included humanitarian, military combat, health-system related and non-human studies.

### Study/source of evidence selection

We exported the retrieved records into a Mendeley Library and subsequently uploaded it to the Rayyan web platform [32] and removed duplicates. Two reviewers (MMA, SS) screened titles and abstracts independently for assessment against the inclusion criteria for the review. Potentially relevant records were retrieved in full, and citation details imported into a Microsoft Excel sheet. A single reviewer (MMA) assessed the full text of selected records in detail against the inclusion criteria. At full-text screening stage, only English (or studies translated into English), and Spanish studies were included. Reasons for exclusion of sources at full text screening stage are reported in the table of excluded studies (see Additional file 1: Appendix C). Any disagreements between the reviewers at each stage were resolved through discussion.

### Data extraction and analysis

A single reviewer (MMA) extracted data from most included records using a data extraction tool (created in Microsoft Excel), assisted by a reviewer extracting the four Spanish records (IF) and checked by another reviewer (SS). This tool was piloted on a small sample of possible included studies, identified in a previous review. Only English and Spanish records were included, as the study was limited in its access to translation services. Data extracted included study types and methodology; characteristics of included CPGs; and AGREE tool use including domain and overall score results. Included synthesis studies were the units of analysis, and simple descriptive statistics was used to conduct the analysis using STATA 14.

Categorical variables were described as percentages; whereas continuous variables were described by means and standard deviations (sd) where data was normally distributed, otherwise reported as median and interquartile range. Data normality was determined graphically and using the skewness-kurtosis test. Studies that calculated median overall scores for AGREE domains were converted to a mean score, as recommended by Hozo et al.      [33]. This allowed one standard summary statistic across domains. Regions were measured according to United Nation Sustainable Development Goals (SDG) regional groupings [34], however other regional groupings were also considered. SDG regions were chosen, due to the meaningful geographical presentation of different regions with comparable income status.

AGREE scores were analysed across subgroups into one of the seven SDG regions. A list of included studies is found in Additional file 1: Appendix D.

## Results

**Fig. 1:**
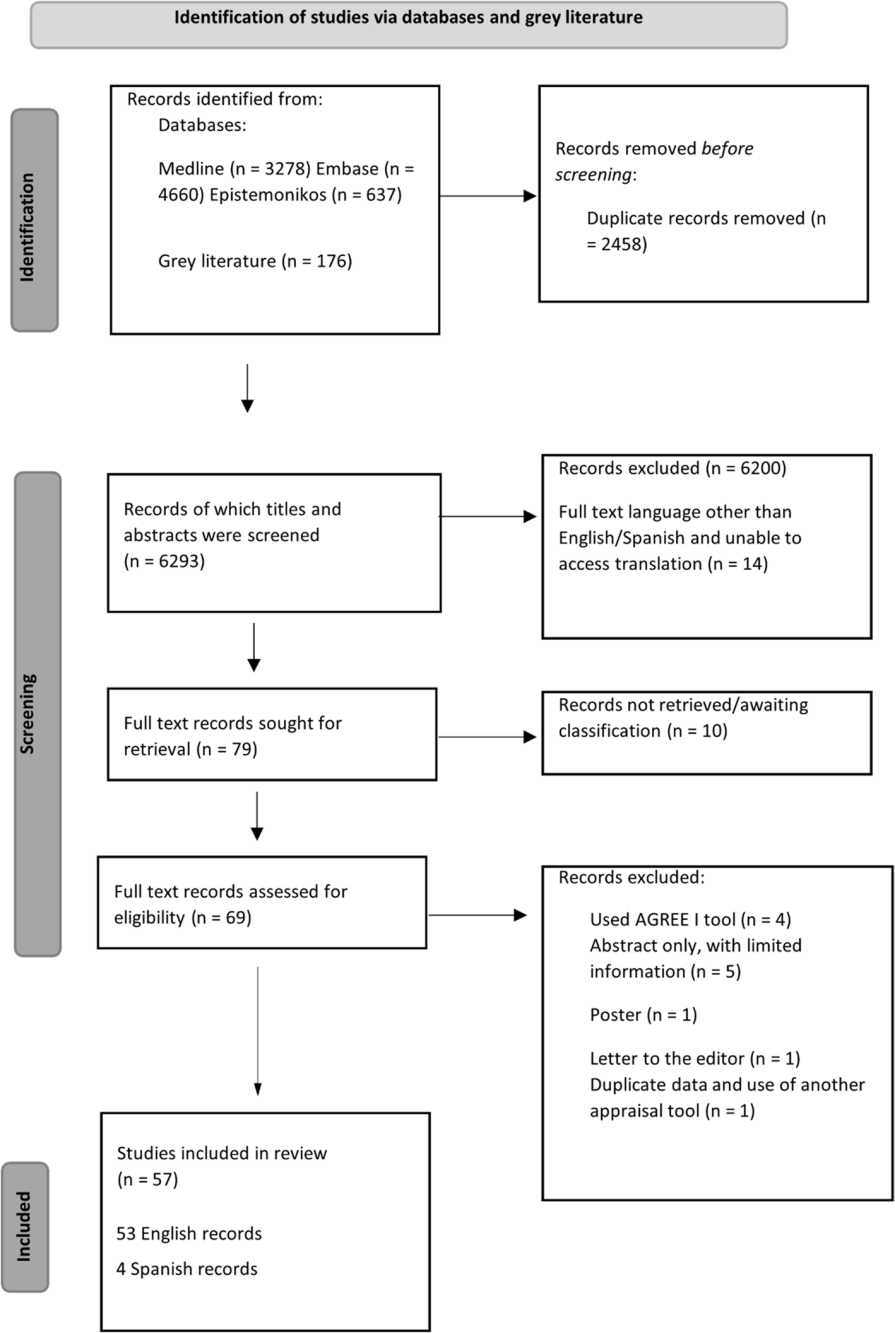
Prisma flow chart of selecting included studies

The results of the search and study inclusion are presented in Fig. 1. A total of 2918 CPGs were included across 57 studies, accounting for all seven SDG regions. Best represented regions were Eastern and South-Eastern Asia, and Europe and Northern America; a well-represented region was Latin America and the Caribbean; whilst least represented areas were Northern Africa and Western Asia, and Oceania. Other regions outside the SDG grouping scope, included; Nordic countries, Asia, Middle East and North Africa, and Africa.

### Characteristics of included studies

Fifty-seven studies were included in the analysis. The median number of included CPGs per study was 20 (IQR 44) and the median year-range of included CPGs was 8.5 years (IQR 6). The general aim of most studies was to describe and determine quality (or lack thereof) of CPGs, in either specific topical areas or country-wide; to enhance future CPG development and/or implementation of current CPGs. This included identifying areas of variability and vulnerability to speak to compliance and conformity. Most studies then used a cross-sectional design and topical purposeful sampling and were published by academic societies or researchers. However, a substantial number (*n* = 14) of studies evaluated all country-wide or regional CPGs. Most studies (*n* = 34, 60%) only searched local/regional databases and grey literature for including CPGs, whilst 20 studies (35%) searched both locally and internationally (including for example, Medline and guideline clearing houses). The number of included studies increased as the year of publication range increased, mostly published after 2019 (Table 1). One study only utilized the overall assessment and did not report domain scores and one study assessed one domain only.

**Table 1:** Broad summary of included studies

Characteristics	n (%)
**Types of studies**	
Cross-sectional analysis	34 (59.6)
Systematic review	17 (29.8)
Review^a^	6 (8.8)
**Year of publication**	
2010 – 2014	9 (15.8)
2015 – 2018	22 (38.6)
2019 – October 2021	26 (45.6)
**Entity responsible for the appraisal**	
Government related	4 (7)
Academic society/Researchers	49 (86)
Professional society	3 (5)
Non-governmental organization	1 (2)
**Geographical locations by SDG region**	
Sub-Saharan Africa	2 (3.7)
Northern Africa and Western Asia	1 (1.8)
Central and Southern Asia	2 (3.7)
Eastern and South-Eastern Asia	20 (38)
Latin America and the Caribbean	9 (17)
Oceania	1 (1.8)
Europe and Northern America	18 (34)
**Sampling of CPGs**	
No sample^b^	14 (24.5)
Random sample	1 (2)
Systematic sample	11 (19)
Topical purposeful sampling	29 (51)
Other purposeful sampling	2 (3.5)

^a^This category broadly includes literature surveys, methodological reviews, narrative reviews, and other non-systematic reviews

^b^All country/regional CPGs were included

### Characteristics of included guidelines

#### Included CPGs

Table 2 illustrates the characteristics of included CPGs indicating that the included guidelines scoped a wide range of topics. Thirty-six studies (63%) did not use a formal definition of what it regarded as a CPG in its inclusion criteria. Subsequently, there was a poor reporting of development methods of CPGs and similarly, use of Grades of Recommendation, Assessment, Development, and Evaluation (GRADE) to assess the certainty of the underlying evidence. Only 10 studies (18%) commented on the methods of development of included studies, where de novo (starting anew) development was the most prevalent (*n* = 5, 50% of the 10 studies). Additionally, overall, only 11 studies (19%) reported the use of GRADE in the development of the included CPGs.

**Table 2:** Characteristics of *n* = 57 included studies and AGREE tool use

Characteristics	n (%)
**Type of included CPGs**	
All national/regional	9 (16)
Governmental	3 (5)
Topical	41 (72)
Professional society	4 (7)
**Methods of CPG development**	
None mentioned	47 (82)
De novo	5 (9)
Adopt	1 (2)
Miscellaneous	4 (7)
**Healthcare topic/area**	
Dermatology	1 (2)
Complementary and alternative medicine	4 (7)
Emergency medicine	1 (2)
Otolaryngology	3 (5)
“High priority diseases”	2 (3.5)
Internal medicine	10 (17)
Neurology	2 (3.5)
Nursing practice	2 (3.5)
Oncology	2 (3.5)
Orthopaedics	4 (7)
Paediatrics	2 (3.5)
Poverty-related	1 (2)
Primary care	1 (2)
Psychiatry	2 (3.5)
Surgery	1 (2)
Mixed	19 (33)
**Strict inclusion criteria regarding the definition of a CPG**	
Yes	21 (37)
No	36 (63)
**AGREE use**	
Used 2–4 assessors	56 (98)
Used overall assessment	43 (74)
Used one overall assessment only	29 (69)
Modified overall assessment	24 (57)

#### AGREE use

AGREE use and completeness, was well reported and presented in all included studies. Most studies used two to four assessors to assess domain scores, and inter-observer agreement scores were used by 36 (63%). Almost three quarters of studies (*n* = 43, 74%) reported the overall guideline assessment; most studies reporting only one overall assessment and modifying it.

#### Overall assessment

The method of formulating the overall quality assessment score of CPGs varied in most of our included studies. The majority of 43 studies (74%) made use of this overall assessment. Twenty-nine studies used one assessment only and thirteen studies used both assessments. However, 24 studies (57%) modified this overall assessment and did not apply it as per the AGREE manual, where 12 variations of modification were found. The three most common modifications included: i) calculating an overall average across the six AGREE domains ii) using a cut-off of *Rigor of development* domain score > 60% and iii) using a cut-off sliding scale of most domain scores > 60% being ‘recommended’, scores of 30–60% ‘recommended with modifications’, and if most scores were < 30% ‘not recommended’. It was noted that even among these three groupings, slight variation existed. Additionally, there was no regional pattern regarding this modification. Latin America and the Caribbean (*n* = 7, 78%), and Europe and Northern America (*n* = 15, 83%), used this overall assessment in most of their studies.

### SDG regions

**Fig. 2:**
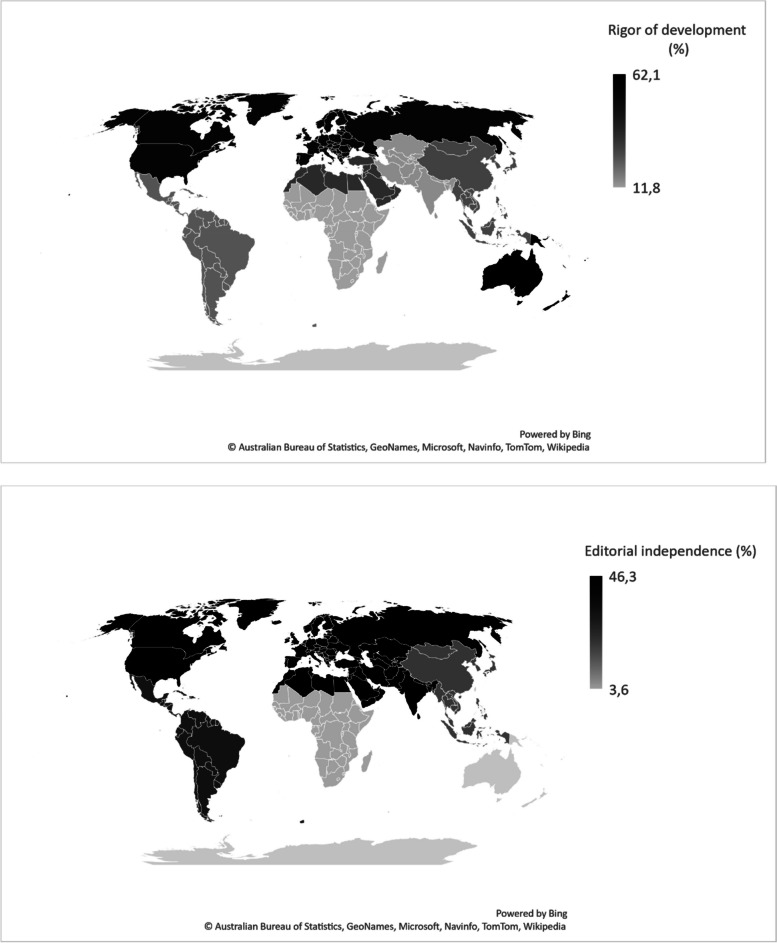
Heatmaps of AGREE domains *Rigor of development* and *Editorial independence* in percentage (%) per SDG region

Overall domain scores were low for *Rigor of development* (ROD), *Applicability*, and *Editorial independence* (EI); moderate for *Stakeholder involvement*; and higher for *Scope and purpose* and *Clarity and presentation* (Table 3). Figure 2 depicts the low global scores for ROD and EI per SDG region; simultaneously noting the low overall range of a maximum of 62.1% for ROD and a maximum of 46.3% for EI.

**Table 3:**
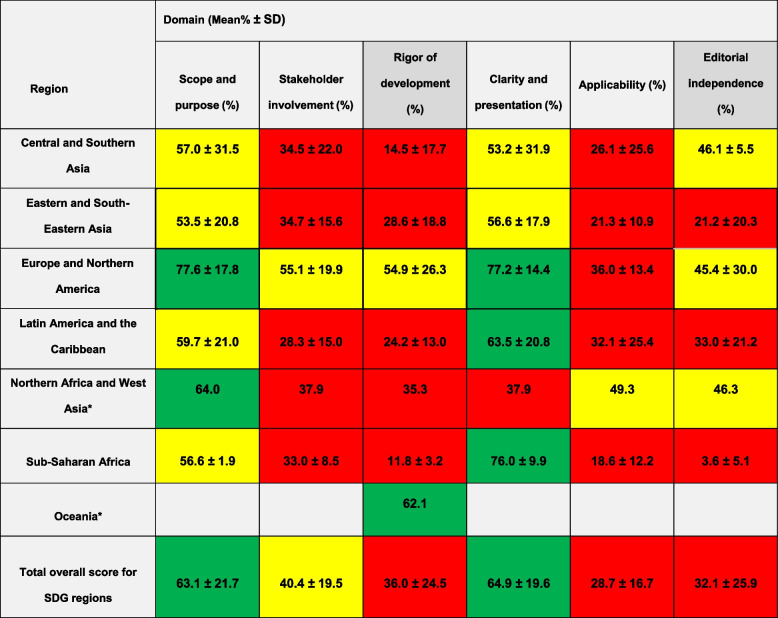
AGREE II domain scores for all SDG regions (*n* = 51)

**Rapid signal for quality of regional CPGs**: Low quality: Red <40%; Moderate quality: Yellow 40%-59%; High quality: Green ≥60%

*Regions only contributing one study, thus no standard deviation derived

**Fig. 3:**
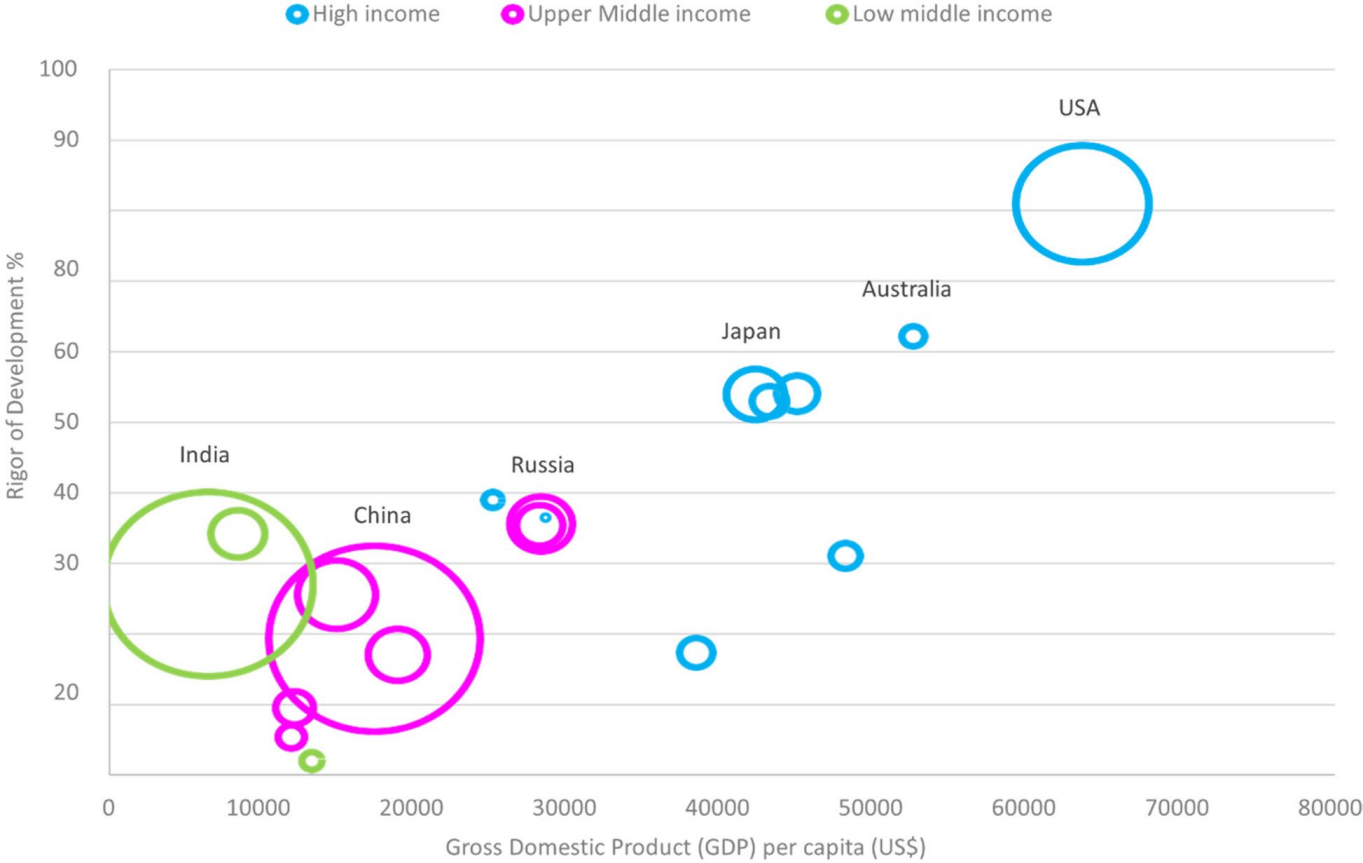
Bubble plot comparing 4 variables, including ROD domain scores, Gross Domestic Product (GDP) per capita, different income-level countries, and population size (size of the bubble)

Figure 3 looks broadly at domain ROD, in relation to Gross Domestic Product (GDP) per capita [35], country-income profile [36], and population size. Here, there is a seemingly linear relationship between the ROD quality score, GDP per capita and country-income status. Lastly, we did not identify any studies representing low-income countries.

## Discussion

### Main findings

This scoping review provided a global view investigating the heterogeneity of CPG synthesis studies across different countries and regions, as noted in other studies [27, 37]. Despite the variation in quality, included CPG synthesis studies (and their included CPGs), were consistently described and adequately assessed and reported with the AGREE tool. This highlights the importance and value of quality assessment tools that are straightforward to use. Characteristics of these synthesis studies, mostly performed by researchers from the academia, were uniformly consisting of cross-sectional reviews where topical sampling was performed. Though high- and middle-income countries and regions were well represented, there were no synthesis studies from low-income countries. Generally, three AGREE domains of *Rigor of development*, *Applicability* and *Editorial independence* had the lowest domain scores, and this was most prevalent in middle-income countries and regions. *Editorial independence* repeatedly scored as the lowest domain, even in countries that had acceptable scores for other domains (including *Rigor of development*). Despite this, many AGREE domains across geography had either moderate or high-quality scores, and the tendency to appraise country-wide or regional CPGs appear to be increasing.

### Characteristics of studies and CPGs

Regions of Eastern and South-Eastern Asia, and Europe and Northern America were the best represented in terms of quantity of studies included. This was recently seen in a living systematic review of Covid-19 rapid guidelines, where 45% of guidelines originated from high income countries (HICs) and no guidelines from low-income countries [38]. The included CPGs were either topical or all country-wide, regional, or governmental CPGs; mainly initiated by the medical society, corresponding to findings of previous reviews [16, 39]. Similar reviews found that high-quality guidelines were more often produced by government-supported organizations and/or within regulated, coordinated programmes [21, 40–42], in comparison to guidelines produced by professional societies.

Regarding the use of GRADE methods, either in evidence synthesis or as part of the evidence to decision process, only 11 synthesis studies (19%) reported using any GRADE methods, two of these primarily assessing GRADE adherence. Lack of reporting of GRADE and other methods of judging the certainty of evidence, along with how evidence is used to develop recommendations, is concerning. Reviewers of CPGs are encouraged to highlight the importance of how these evidence-to-decision processes evolve, to strengthen guideline development and assessment in the future. Regional quality assessments have shown that there is a slow but increasing incorporation of these requirements [43, 44]. Online tools, such as GRADEpro [45], can make the process transparent, faster, and more user-friendly in a fast-paced age.

Reciprocally, the low reporting rate of guideline development methods and absence of Covid-19 or rapid guidelines included in this scoping review, could signify areas of much needed growth. Through multiple online high-quality/tech savvy platforms created for the Covid pandemic, the guideline community can be inspired to make use of these types of resources to further advance guideline quality. This can assist in processes like guideline adaptation, that aim to reduce research waste and duplication of effort; by using living reviews and guideline repositories [46], recommendation mapping [47], and guideline development resources such as produced by e-COVID [48]. A future research focus on adaptation methodology such as GRADE Adolopment and others [49], can be further supported in LMICs to build capacity for guideline development in these regions.

The Inclusive Internet Index is an example of a starting point to plug LMICs into these resources. As the 2021 report [50] indicates, there is still a ‘digital divide’ preventing most LMICs from accessing adequate internet supplies, where sub-Saharan Africa was shown to have the most constrained connectivity globally. Future research can involve investigating the challenges of LMICs on internet connectivity, especially regarding uptake and implementation of CPGs. This can include investigating the uptake of evidence-to-decision online tools, and how this translates into CPG quality.

### AGREE tool use and domains

AGREE tool use was well reported where most synthesis studies used a recommended two to four assessors per CPG, reported on interobserver agreement and assessed all 6 domains; similar to previous reviews [16, 39]. The idea of rating the overall quality of CPGs has been the subject of much debate. The AGREE tool recommends focusing on all six domains and not solely using the overall assessment to judge quality. Several authors have suggested different thresholds for this overall score, mostly suggesting using the strength of the *Rigor of development* domain. Two recent studies attempted rating the importance of the different AGREE domains on the overall assessment, indicating that *Rigor of development*, *Clarity of presentation*, *Applicability*, and *Editorial independence* had a significant influence on the overall quality assessment [37, 51]. Almost three quarters (74%) of the synthesis studies included in this scoping review, used this overall assessment to define its CPGs’ quality, and this is similar to the finding of two other publications [51, 52]. Those opting to use this overall assessment used multiple ways to modify it, similar to a review where only 23% of included studies provided clear criteria for generating the cut-offs applied [53]. This could indicate that several users would welcome an explicit distinction between high- and low-quality guidelines [5]. The important domain of *Rigor of development*, particularly used in the overall CPG quality consideration, is often scored as low [8, 10, 21], similarly found in this study. Notably, domain *Editorial independence* is becoming increasingly scrutinized as important for guideline quality [24]. In fact, Molino et al. showed that higher quality CPGs reported its funding sources 10 times more often than those of lower quality [21]. Multiple recent studies reaffirmed the low scores across guidelines for this domain [54–56]; and this gap is crucial in LMICs especially [57], however, it can be argued that it is equally important for all countries world-wide.

### Strengths and limitations

The scoping review design used in this study allowed us to attain a global overall view. This review focused on studies that used the AGREE tool for quality assessment and cannot comment on synthesis studies of guidelines using other appraisal tools. Additionally, there might be included studies that applied AGREE to other types of documents (such as end-user guidance documents or broad summary documents). There has been an indication that other types of ‘guidance documents’ have lower AGREE scores than true CPGs [58], and this could have overestimated the results of this study. This scoping review cannot conclude that CPGs from HICs are of higher quality than those of LMICs, as we are limited by the availability of studies summarizing the available guidelines per regions. In addition to this, the CPGs assessed for each region was often a sub-sample of all available CPGs in that region. Importantly, regional or national CPG synthesis studies, may be preferentially submitted to local journals, without indexing in international databases. Subsequently, these researchers may be inclined to not publish at all, simply posting results on association or organizational websites, resulting in potential missed studies. Cumulatively, this could have influenced the results of this scoping review, especially the associations with world regions and income groups, as LMICs could be more inclined to solely make use of local distributions of quality assessments of CPGs.

## Conclusion

When looking at the landscape of guideline quality, there has been various attempts to level the playfield and inroads have been made. There is a current tendency to critically evaluate guideline quality in country-wide and regional approaches and AGREE is overarchingly used well in this practice. However, guideline *Rigor of development* varies between HICs and LMICs, necessitating building further guideline development capacity, including use of GRADE for guidelines. Improved reporting of funding and competing interests, as well as guideline development approaches and their underlying evidence sources, can further enhance regional quality of guidelines. Assessing country-wide or regional guidelines with quality appraisal tools, could advance overall guideline quality for all areas globally. This is an important step forward and toward global guideline uniformity, as clinicians are in dire need of high-quality guidelines to improve delivery and quality of care.

### Supplementary Information

**Additional file 1.**
