# Supplementary material for: Advancing guideline quality through country-wide and regional quality assessment of CPGs using AGREE: a scoping review
Source: BMC Med Res Methodol. 2023 Nov 30;23:283. doi: 10.1186/s12874-023-02101-5 (PMC10690993; doi:10.1186/s12874-023-02101-5)
Supplement: Supplementary file 1 — Additional file 1. [file 12874_2023_2101_MOESM1_ESM.docx]

# Supplementary file: Appendices

**Appendix B: Search strategies**

Medline PubMed search strategy

#1 "Practice Guideline"[Publication Type] #2 "Practice Guidelines as Topic"[Mesh]

#3 “practice guideline”[Title/Abstract] OR “practice guidelines”[Title/Abstract] #4 CPGs[Title/Abstract] OR CPG[Title/Abstract]

#5 #1 OR #2 OR #3 OR #4

#6 "agree II"[Title/Abstract] OR "Appraisal of Guidelines for Research and Evaluation"[Title/Abstract] OR "Appraisal of Guidelines for Research and Evaluation II"[Title/Abstract] OR "Agree 2"[Title/Abstract] OR "AGREE-REX"[Title/Abstract] OR "AGREE GRS"[Title/Abstract] OR "appraise"[Title/Abstract] OR "appraisal"[Title/Abstract] OR "appraised"[Title/Abstract] OR "quality assessment"[Title/Abstract] OR "quality appraisal"[Title/Abstract] OR "quality evaluation"[Title/Abstract] OR "evaluation of quality"[Title/Abstract]

#7 #5 AND #6

Embase search strategy

**Embase**1974 to 2021 October 01

1. exp practice guideline/

2. practice guideline$.tw.

3. CPG$.tw.

4. or/1-3

5. (AGREE tool or AGREE-tool or (Appraisal of Guidelines for Research and Evaluation)).tw.

6. (AGREEII or AGREE II or AGREE-II or (Appraisal of Guidelines for Research and Evaluation)).tw.

7. (AGREE-GRS or AGREE GRS or AGREEGRS or (AGREE adj3 global rating scale)).tw.

8. (AGREE-REX or AGREE REX or AGREEREX or (AGREE adj3 Recommendations excellence)).tw.

9. (quality adj2 (assessment or apprais$ or evaluation$)).tw.

10. 5 or 6 or 7 or 8 or 9

11. 4 and 10

**Appendix C: List of excluded studies**

| **Study title** | **Reason for exclusion** |
| --- | --- |
| Stoffer, *et al* (2012) | Meeting abstract |
| Piano, *et al* (2012) | Poster |
| Hu, *et al* (2013) | Used AGREE I |
| Brenol, *et al* (2015) | AGREE I used for commenting on CPG development. Tool not used to appraise. Survey. |
| Sampson, *et al* (2015) | Used AGREE I |
| Laranjeira, *et al* (2016) | Abstract |
| Grimmer, *et al* (2016) | AGREE tool compared to iCAHE. Duplicate of data of included study nr 20 |
| Jiang *et al* (2017) | Abstract |
| Borgonjen *et al* (2017) | Letter to the editor |
| Li *et al* (2018) | Abstract |
| Seto *et al* (2019) | Used AGREE I |
| Yamashita, H (2020) | Abstract |

**Appendix D: List of included studies**

| Author, year | Title | Design | Country or Region | Government/ society | Sampling |
| --- | --- | --- | --- | --- | --- |
| 1. Kredo et al, 2012 | Clinical practice guidelines within the Southern African development community: a descriptive study of the quality of guideline development and concordance with best evidence for five priority diseases | Cross-sectional | SADC | Academic society/ Researchers | Systematic |
| 1. Chen et al, 2012 | Quality assessment of clinical guidelines in China: 1993 - 2010 | Systematic review | China | Academic society/ Researchers | All country/regional CPGs |
| 1. Tudor et al, 2013 | Methodological Rigour and Transparency of Clinical Practice Guidelines Developed by Neurology Professional Societies in Croatia | Cross-sectional | Croatia | Professional society | Topical |
| 1. Zhang et al, 2013 | Analysis of Quality of Clinical Practice Guidelines for Otorhinolaryngology in China | Cross-sectional | China | Academic society/Researchers | Topical |
| 1. Isaac et al, 2013 | Quality of Reporting and Evidence in American Academy of Pediatrics Guidelines | Cross-sectional | North America | Academic society/Researchers | All country/regional CPGs |
| 1. Piano et al, 2012 | Guidelines for Neuropathic Pain Management in Patients with Cancer: A European Survey and Comparison | Cross-sectional | Europe | Academic society/Researchers | Topical |
| 1. Yuwen et al, 2014 | Appraisal of clinical practice guidelines for the management of rheumatoid arthritis in traditional Chinese medicine using the AGREE II instrument: A systematic review | Systematic review | China | Academic society/Researchers | Systematic |
| 1. Jiang et al, 2015 | Quality Assessment of Clinical Practice Guidelines for Respiratory Diseases in China | Cross-sectional | China | Academic society/Researchers | Topical |
| 1. Sabharwal et al, 2014 | High methodologic quality but poor applicability: assessment of the AAOS guidelines using the AGREE II instrument | Cross-sectional | North America | Professional society | Topical |
| 1. Hester et al, 2014 | Methodological quality of national guidelines for pediatric inpatient conditions | Cross-sectional | North America | Academic society/Researchers | Topical |
| 1. Yaman et al, 2015 | A critical appraisal of the North American Spine Society guidelines with the Appraisal of Guidelines for Research and Evaluation II instrument | Cross-sectional | North America | Professional society | Topical |
| 1. Choi et al, 2015 | The quality of clinical practice guidelines in traditional medicine in Korea: appraisal using the AGREE II instrument | Review | Korea | Academic society/Researchers | Topical |
| 1. Chen et al, 2015 | Clinical practice guidelines for hypertension in China: a systematic review of the methodological quality | Systematic review | China | Academic society/Researchers | Topical |
| 1. Molino et al, 2016 | Non-Communicable Disease Clinical Practice Guidelines in Brazil: A Systematic Assessment of Methodological Quality and Transparency | Cross-sectional | Brazil | Academic society/Researchers | Systematic |
| 1. Yaşar et al, 2016 | Quality Assessment of Clinical Practice Guidelines Developed by Professional Societies in Turkey | Cross-sectional | Turkey | Academic society/Researchers | Systematic |
| 1. Werner et al, 2015 | The quality of European dermatological guidelines: critical appraisal of the quality of EDF guidelines using the AGREE II instrument | Cross-sectional | Europe | NGO | All country/regional CPGs |
| 1. Jin et al, 2016 | Nursing Practice Guidelines in China do Need Reform: A Critical Appraisal Using the AGREE II Instrument | Cross-sectional | China | Academic society/Researchers | Systematic |
| 1. Chang et al, 2016 | Methodological Quality Appraisal of 27 Korean Guidelines Using a Scoring Guide Based on the AGREE II Instrument and a Web-based Evaluation | Cross-sectional | Korea | Academic society/Researchers | Convenience |
| 1. Canelo-Aybar et al, 2016 | Clinical practice guidelines in Peru: evaluation of its quality using the AGREE II instrument | Cross-sectional | Peru | Academic society/Researchers | All country/regional CPGs |
| 1. Tang et al, 2017 | A concise review of current guidelines for the clinical management of hepatocellular carcinoma in Asia | Review | Asia | Academic society/Researchers | Topical |
| 1. Martin‐Galindo et al, 2017 | European Fissure Sealant Guidelines: assessment using AGREE II | Cross-sectional | Europe | Academic society/Researchers | Topical |
| 1. Machingaidze et al, 2016 | Quality and Reporting Standards of South African Primary Care Clinical Practice Guidelines | Cross-sectional | South-Africa | Academic society/Researchers | Convenience |
| 1. Yao et al, 2017 | Appraising the quality of clinical practice guidelines in traditional Chinese medicine using AGREE II instrument: A systematic review | Systematic review | China | Academic society/Researchers | Topical |
| 1. Yao et al, 2016 | Quality Assessment of Clinical Practice Guidelines for Integrative Medicine in China: A Systematic Review | Systematic review | China | Academic society/Researchers | Topical |
| 1. O'Donoghue et al, 2017 | Systematic review of clinical practice guidelines in kidney transplantation | Systematic review | UK | Academic society/Researchers | Topical |
| 1. Lienhard et al, 2018 | Assessing methodological quality of Russian clinical practice guidelines and introducing AGREE II instrument in Russia | Cross-sectional | Russia | Academic society/Researchers | Systematic |
| 1. Rodríguez et al, 2016 | Quality assessment of clinical practice guidelines of the Chilean explicit guarantees in healthcare program | Cross-sectional | Chile | Academic society/Researchers | All country/regional CPGs |
| 1. Parra-Anguita et al, 2016 | Quality of the Spanish Clinical Guidelines about Alzheimer's Disease and others Dementias | Cross-sectional | Spain | Academic society/Researchers | All country/regional CPGs |
| 1. Timaná et al, 2018 | Characteristics and quality of the of clinical practice guidelines in the Social Security of Health of Peru | Cross-sectional | Peru | Academic society/Researchers | Random |
| 1. Bhaumik et al, 2018 | Clinical Practice Guidelines in India: quality appraisal and the use of evidence in their development | Cross-sectional | India | Academic society/Researchers | Systematic |
| 1. Yuwen et al, 2018 | Appraisal of the Quality and Contents of Clinical Practice Guidelines for Hypertension Management in Chinese Medicine: A Systematic Review | Review | China | Academic society/Researchers | Topical |
| 1. Costa Molino et al, 2019 | Comparison of the methodological quality and transparency of Brazilian practice guidelines | Review | Brazil | Academic society/Researchers | Systematic |
| 1. Okwen et al, 2019 | Evaluation of all African clinical practice guidelines for hypertension: Quality and opportunities for improvement | Review | Africa | Academic society/Researchers | Topical |
| 1. Talagala et al, 2019 | Sri Lankan clinical practice guidelines: A methodological quality assessment utilizing the AGREE II instrument | Cross-sectional | Sri Lanka | Academic society/Researchers | Random |
| 1. Gao et al, 2019 | Quality appraisal of clinical practice guidelines for diabetes mellitus published in China between 2007 and 20Cross-sectional7 using the AGREE II instrument | Systematic review | China | Academic society/Researchers | Topical |
| 1. Jarl et al, 2019 | Nordic clinical guidelines for orthotic treatment of osteoarthritis of the knee: A systematic review using the AGREE II instrument | Systematic review | Nordic region | Academic society/Researchers | Topical |
| 1. Hatakeyama et al, 2019 | The structure of the quality of clinical practice guidelines with the items and overall assessment in AGREE II: a regression analysis | Cross-sectional | Japan | Academic society/Researchers | All country/regional CPGs |
| 1. Sasaki et al, 2019 | Updates to and quality of clinical practice guidelines for high-priority diseases in Japan | Cross-sectional | Japan | Governmental | Topical |
| 1. Li et al, 2021 | Status Quo and Analysis of the Cardiovascular Clinical Practice Guidelines/Expert Consensuses of Chinese and Integrative Medicine: A Systematic Review | Systematic review | China | Academic society/Researchers | Topical |
| 1. Rodríguez et al, 2020 | Plasmodium vivax malaria across South America: management guidelines and their quality assessment | Systematic review | South America | Academic society/Researchers | Topical |
| 1. Medina et al, 2019 | European clinical practice guidelines for depression in adults: Are they good enough? | Cross-sectional | European | Academic society/Researchers | Topical |
| 1. Santana et al, 2018 | The different clinical guideline standards in Brazil: High-cost treatment diseases versus poverty-related diseases | Cross-sectional | Brazil | Governmental | All country/regional CPGs |
| 1. Zupon et al, 2019 | An appraisal of emergency medicine clinical practice guidelines: Do we agree? | Cross-sectional | North America | Academic society/Researchers | Topical |
| 1. Suzuki et al, 2020 | Clinical practice guidelines for acute otitis media in children: a systematic review and appraisal of European national guidelines | Systematic review | European | Academic society/Researchers | Topical |
| 1. Wang et al, 2020 | The methodology for developing nursing clinical practice guidelines over recent decades in China: A critical appraisal using AGREE II | Review | China | Academic society/Researchers | Topical |
| 1. Mir et al, 2020 | Management of rectal cancer in Canada: an evidence-based comparison of clinical practice guidelines | Cross-sectional | Canada | Academic society/Researchers | Topical |
| 1. Flynn et al, 2020 | Appraising Otolaryngology–Head and Neck Surgery Clinical Practice Guidelines for Effective Dissemination and Implementation Design | Cross-sectional | North America | Academic society/Researchers | Topical |
| 1. Loezar et al, 2020 | Guidelines in Low- and Middle-Income Countries Paper 2: Quality assessment of Chilean guidelines: need for improvement in rigor, applicability, updating, and patients’ inclusion | Cross-sectional | Chile | Academic society/Researchers | All country/regional CPGs |
| 1. Dans et al, 2020 | Guidelines in Low- and Middle-Income Countries Paper 3: Appraisal of Philippine Clinical Practice Guidelines using Appraisal of Guidelines for Research and Evaluation II: improvement needed for rigor, applicability, and editorial independence | Cross-sectional | Philippines | Governmental | All country/regional CPGs |
| 1. Corp et al, 2021 | Evidence-based treatment recommendations for neck and low back pain across Europe: A systematic review of guidelines | Systematic review | European | Academic society/Researchers | Systematic |
| 1. Ciquier et al, 2020 | Assessing the quality of seven clinical practice guidelines from four professional regulatory bodies in Quebec: What's the verdict? | Cross-sectional | Quebec | Academic society/Researchers | Convenience |
| 1. Almazrou et al, 2021 | Assessing the Quality of Clinical Practice Guidelines in the Middle East and North Africa (MENA) Region: A Systematic Review. | Systematic review | Middle East and North Africa | Academic society/Researchers | All country/regional CPGs |
| 1. Colunga-Lozano et al, 2020 | Methodological assessment of Mexican Clinical Practice Guidelines: GRADE framework adherence and critical appraisal | Cross-sectional | Mexico | Academic society/Researchers | All country/regional CPGs |
| 1. Zhou et al, 2021 | Clinical Epidemiology in China series. Paper 4: The reporting and methodological quality of Chinese clinical practice guidelines published between 20Cross-sectional4 and 20Cross-sectional8: A systematic review | Systematic review | China | Academic society/Researchers | Systematic |
| 1. Wan et al, 2021 | Quality changes of clinical practice guidelines for respiratory diseases in China: A systematic review | Systematic review | China | Academic society/Researchers | Topical |
| 1. Kataoka et al, 2021 | Quality of clinical practice guidelines in Japan remains low: A cross-sectional meta-epidemiological study | Cross-sectional | Japan | Academic society/Researchers | Systematic |
| 1. Barker et al, 2021 | Guidelines rarely used GRADE and applied methods inconsistently: A methodological study of Australian guidelines | Review | Australia | Academic society/Researchers | All country/regional CPGs |
